# 3,2′-Dihydroxyflavone Improves the Proliferation and Survival of Human Pluripotent Stem Cells and Their Differentiation into Hematopoietic Progenitor Cells

**DOI:** 10.3390/jcm9030669

**Published:** 2020-03-02

**Authors:** Kyeongseok Kim, Ahmed Abdal Dayem, Minchan Gil, Gwang-Mo Yang, Soo Bin Lee, Oh-Hyung Kwon, Sangbaek Choi, Geun-Ho Kang, Kyung Min Lim, Dongho Kim, Ssang-Goo Cho

**Affiliations:** 1 Department of Stem Cell & Regenerative Biotechnology and Incurable Disease Animal Model and Stem Cell Institute (IDASI), Konkuk University, Seoul 05029, Korea; proproggs@naver.com (K.K.); ahmed_morsy86@yahoo.com (A.A.D.); minchangil@gmail.com (M.G.); slayersgod@nate.com (G.-M.Y.); soobineey@naver.com (S.B.L.); hjyone@naver.com (S.C.); geunhokang@naver.com (G.-H.K.); lmin0217@naver.com (K.M.L.); 2 Bio-Medical Science (BMS) Co., Ltd., Gimpo 10136, Korea; oh-kwon@lascience.co.kr (O.-H.K.);

**Keywords:** flavonoid, 3,2′-DHF, naïve hiPSC, single cell aneuploidy screening, RNA sequencing, FreSHtracer, hematopoietic stem cell differentiation, natural killer cells

## Abstract

Efficient maintenance of the undifferentiated status of human pluripotent stem cells (hiPSCs) is crucial for producing cells with improved proliferation, survival and differentiation, which can be successfully used for stem cell research and therapy. Here, we generated iPSCs from healthy donor peripheral blood mononuclear cells (PBMCs) and analyzed the proliferation and differentiation capacities of the generated iPSCs using single cell NGS-based 24-chromosome aneuploidy screening and RNA sequencing. In addition, we screened various natural compounds for molecules that could enhance the proliferation and differentiation potential of hiPSCs. Among the tested compounds, 3,2′-dihydroxyflavone (3,2′-DHF) significantly increased cell proliferation and expression of naïve stemness markers and decreased the dissociation-induced apoptosis of hiPSCs. Of note, 3,2′-DHF-treated hiPSCs showed upregulation of intracellular glutathione (GSH) and an increase in the percentage of GSH-high cells in an analysis with a FreSHtracer system. Interestingly, culture of the 3,2′-DHF-treated hiPSCs in differentiation media enhanced their mesodermal differentiation and differentiation into CD34+ CD45+ hematopoietic progenitor cells (HPC) and natural killer cells (NK) cells. Taken together, our results demonstrate that the natural compound 3,2′-DHF can improve the proliferation and differentiation capacities of hiPSCs and increase the efficiency of HPC and NK cell production from hiPSCs.

## 1. Introduction

Pluripotent stem cells (PSCs) can be classified into embryonic stem cells (ESCs), which are derived from the inner cell mass of blastocyst-stage embryos, and induced pluripotent stem cells (iPSCs), which can be generated by introducing pluripotency factors such as Oct4, Sox2, and Klf4 [1,2]. PSCs are valuable resources in developmental biology, disease modeling, and regenerative medicine [3,4], and the preparation of PSCs with improved proliferation, survival, self-renewal, and differentiation capacities is important for successful application of PSCs in stem cell research and therapy [5].

As an important application of hiPSCs, combining iPSC technology with hematopoietic differentiation protocols has the potential to produce a multitude of histocompatible hematopoietic progenitor cells (HPCs) and immune cells for clinical uses, such as bone marrow transplantation or cancer therapy [6,7]. The genetic manipulation of hiPSCs and immune cells to prepare chimeric antigen receptor (CAR)-T or CAR-NK cells has significant potential as a new cell-mediated immunotherapy for intractable malignant tumors [8]. In particular, NK cells are a group of lymphocytes that play an important role in the innate immune system and their extensive cytotoxicity and rapid killing capacities make them ideal for use in cancer immunotherapy [9,10].

The interactive network of transcription factors, such as Oct4, Nanog, and Sox2, modulates the molecular control of pluripotency and self-renewal in PSCs. These transcription factors are uniquely expressed in PSCs to orchestrate the transcriptional regulation of pluripotency by collaboratively activating the transcription of one another, thus constituting an auto-regulatory circuit [11,12], and are responsible for driving the expression of genes essential to pluripotency and self-renewal [12,13]. Accordingly, the discovery of novel compounds that regulate this complex transcriptional network in stem cells is important to produce PSCs with improved proliferation, survival and differentiation capacities.

Flavonoids are natural compounds, which are widely dispersed in plant pigments and are present in a wide range of foods [14,15,16]. We have previously reported an array of flavonoid molecular functions in vitro and in vivo, including anti-apoptotic [17,18,19], antiviral [20,21], and antidiabetic [22] activities, adipogenesis modulation [23], pluripotency marker expression and various neuroprotective properties [24]. Of note, the differential action of the flavonoids in various cell lines is attributed to their chemical structure and the location of the functional groups within the benzene rings. Our study showed the impact of the changes in the position of the functional groups within the B ring of the diphenylpropane (C_6_C_3_C_6_) skeleton on the molecular functions of flavonoids in various cell lines [17,25,26,27]. Here, we screened flavonoids with differential hydroxylation of the flavonoid B ring for compounds that enhanced hiPSC proliferation and found that 3,2′-dihydroxyflavone (3,2′-DHF) improved both proliferation and stemness of hiPSCs. Interestingly, 3,2′-DHF treatment promoted the naïve state of PSCs, reduced dissociation-induced apoptosis, and increased the percentage of GSH-high cells. Importantly, 3,2′-DHF treatment enhanced the production of PSC-derived HPCs and NK cells. Collectively, these findings show that 3,2′-DHF can be used to produce hiPSCs with improved proliferation, survival, and differentiation capacities for clinical therapeutic applications.

## 2. Materials and Methods

### 2.1. Preparation of Peripheral Blood Mononuclear Cells (PBMCs), hESCs and Generation of hiPSCs

For iPSC generation, the human PBMCs were prepared from blood obtained from healthy donors or patients as follows: after receiving the approval of the institutional review board (IRB) at Konkuk University Bioethics Committee (IRB 7001355-201507-BR-072), PBMCs were prepared from the blood of healthy donors for iPSC generation using density gradient centrifugation with a Ficoll-Paque PLUS (GE Healthcare, Chicago, IL, USA). Briefly, 10 mL of a heparinized blood was mixed with 25 mL of phosphate-buffered saline (PBS). Then, this mixture was carefully added to another tube containing 15 mL of Ficoll-Paque PLUS and then subjected to the centrifugation at 400 g for 30 min at 20 °C. The PBMCs between the top layer and the middle suspension were transferred to a new 15 mL conical tube. Next, we added 5 mL of PBS to the isolated PBMCs and the cells were centrifuged at 300 g for 10 min at 20 °C. Afterwards, the supernatant was carefully removed and the washing procedure using PBS was repeated twice. The suspension of the isolated PBMCs was maintained using Iscove′s Modified Dulbecco′s Medium (IMDM, Thermo Fisher Scientific, Waltham, MA, USA) with 20% knockout serum replacement (KOSR, Thermo Fisher Scientific), 1% penicillin/streptomycin (P/S, Thermo Fisher Scientific) and cytokines, such as stem cell factor (SCF, PeproTech, Rocky Hill, NJ, USA), FMS-like tyrosine kinase 3 ligand (FLT3L, PeproTech), interleukin 3 (IL-3, PeproTech), and interleukin 6 (IL-6, PeproTech).

Generation of hiPSCs (human dermal fibroblast derived iPSC – DF-iPSC), human urine stem cell derived iPSC (U-iPSC) and human PBMC-derived iPSC (PBMC-iPSC, PB-iPSC) was carried out through the transduction of reprogramming vectors of the Yamanaka factors, Oct4, Sox2, Klf4, and c-Myc with a CytoTune-iPS 2.0 Sendai Reprogramming Kit (Life Technologies, Carlsbad, CA, USA). PBMCs were plated in Matrigel-coated six-well culture dishes 2 days before the transduction. On day 0, cells were transduced with Sendai virus (SeV) at the appropriate multiplicity of infection (MOI). The medium for the transduced cells was changed 2 days later and every 2 days thereafter, the mTeSR1 medium was replaced until colonies formed. For PBMCs, 4 days before the transduction, 5 × 10^5^ cells per well were plated in twenty-well culture dishes containing IMDM with 20% KOSR, 1% P/S, and cytokines. On day 0, cells were transduced with SeV at the appropriate MOI overnight. Transduced cells were plated onto a six-well plate culture with mitomycin C (MMC)-treated mouse embryo fibroblast (MEF) feeders in iPSC media (KnockOut™ DMEM (Dulbecco’s Modified Eagle Medium)/F-12 (Thermo Fisher Scientific) supplemented with 20% knockout serum replacement (Thermo Fisher Scientific), 1 mM nonessential amino acids (Thermo Fisher Scientific), 1 mM GlutaMax (Thermo Fisher Scientific), and 1% penicillin/streptomycin (Thermo Fisher Scientific)). Finally, on day 8, the iPSC medium was replaced with iPSC medium supplemented with basic fibroblast growth factor (bFGF) until colonies formed. The hESC line (SNUhES31) were obtained from the Korea Centers for Disease Control & Prevention (KCDC, IRB 7001355-201809-LR-267).

### 2.2. Culture of the Primed State or Naïve State hiPSCs

The primed state hiPSCs were maintained under feeder-free conditions using Matrigel (BD Biosciences, Franklin Lakes, NJ, USA) and mTeSR1 media (STEMCELL Technologies, Vancouver, BC, Canada). When cell confluency reached 80–90%, iPSC colonies were dissociated in clusters using Accutase at 37 °C for 5 min and then cultured in mTeSR1 media supplemented with or without 10 μM ROCK inhibitor, Y-27632 (STEMCELL Technologies) and 3,2′-DHF.

To derive naïve state hiPSCs, the culture media for the primed iPSCs were changed into naïve state conversion media [28] based on mTeSR1 supplemented with PD0325901 (MEK1/2 inhibitor, Sigma, St. Louis, MO, USA, 0.5 μM), CHIR99021 (GSK-3 inhibitor, Sigma, 3 μM), forskolin (Adenylyl cyclase activator, Sigma, 10 μM), and leukemia inhibitory factor (LIF) recombinant human protein (Thermo Fisher Scientific, 10 ng/mL) (2iFL). Lysophosphatidic acid (LPA) (Sigma, 10 μM) was added to naïve state conversion media with ROCK inhibitor, Y-27632, during the subculture and naïve state, hiPSCs were maintained on Matrigel-coated plates. For passaging the naïve hiPSCs, cells were washed once with PBS and gently dissociated to single cells with StemPro Accutase (Thermo Fisher Scientific) and seeded again onto Matrigel-coated plates.

### 2.3. Single Cell Sequencing for Chromosome Aneuploidy Screening 

hiPSCs were cultured in 35 mm culture dishes coated with Matrigel using mTeSR1 media for 3 days. Cells were collected in 15 mL tubes (SPL), centrifuged at 1000 rpm for 5 min, and re-suspended in 1 mL PBS. Re-suspended single cells were harvested using pico-pipet (NEPAGENE, Ichikawa, Japan), and transferred into sterile 0.2 mL PCR tubes. The whole-genome amplification (WGA) and single cell next-generation sequencing (NGS)-based karyotypic analyses were performed by BMS Corporation (Gimpo, Republic of Korea).

For WGA, cell samples were collected in 2 μL PBS, lysed with 2 μL SurePlex cell extraction buffer, and incubated with 5 μL SurePlex Extraction cocktail master mix at 75 °C for 10 min, followed by further incubation at 95 °C for 4 min. Then, genomic DNA (gDNA) was fragmented by the addition of 5 μL SurePlex pre-amplification cocktail to the lysed samples, and the mixture was incubated according to the following protocol: one cycle of 95 °C for 2 min, followed by 12 cycles of 95 °C for 15 s, 15 °C for 50 s, 25 °C for 40 s, 35 °C for 30 s, 65 °C for 40 s, and 75 °C for 40 s, followed by a hold at 4 °C. Subsequently, gDNA was amplified using the PicoPLEX WGA Kit (New England Biolabs (NEB), Ipswich, MA, USA) according to the following thermal cycler program: 1 cycle of 95 °C for 2 min, followed by 15 cycles of 95 °C for 15 s, 65 °C for 1 min and 75 °C for 1 min, followed by a keep at 4 °C. To determine the success of the amplification, 5 μL of each amplified sample and 5 μL of gel loading buffer were used for electrophoretic analysis in a 1.5 % agarose TBE gel.

Amplified samples for NGS were processed with VeriSeq PGS kit (Illumina, San Diego, CA, USA). Library preparation was performed using the Nextera XT 96-Index Kit (Illumina). Briefly, amplified samples were diluted, and concentration was measured with Qubit dsDNA HS assay kit (Life Technologies). One nanogram of total amplified DNA (0.2 ng/mL) of each sample was tagmented (tagged and fragmented) by the VeriSeq PGS transposome according to the manufacture′s protocol. The tagmentation step was carried out at 55 °C for 5 min and held at 10 °C. The resulting tagmented mixture was neutralized by adding 5 μL of proprietary neutralization buffer. After homogenization, the tagmentation plate was held at 20 to 25 °C for 5 min.

The fragmented DNA was amplified with index primer (i7) and (i5) to become the NGS library via a limited-cycle PCR program (1 cycle of 72 °C for 3 min, followed by 12 cycles of 95 °C for 10 s, 55 °C for 30 s and 72 °C for 30 s, 1 cycle at 72 °C for 30 s, followed by a keep at 4 °C). Finally, all libraries were pooled and loaded into the VeriSeq PGS sequencing cartridge following the manufacture′s protocol. The NGS library was sequenced with an Illumina MiSeq system and sequencing data were generated by MiSeq Reporter Software. The following bioinformatic analysis was accomplished with a pre-release version of BlueFuse Multi for NGS (Illumina). Each chromosome was divided into intervals covering approximately 1 Mb of sequence each. Filtered reads from each sample were then mapped into the corresponding chromosome interval or bin.

### 2.4. RNA Sequencing and Gene Ontology (GO) Analysis

For RNA sequencing (RNA Seq) analysis, cells were trypsinized, centrifuged, and rinsed once with ice-cold PBS and total RNA was extracted using TRIzol RNA Isolation Reagents (Life Technologies). The quantity and quality of the total RNA were evaluated using an Agilent 2100 bioanalyzer RNA kit (Agilent, Santa Clara, CA, USA). The isolated total RNA was prepared for processing with an mRNA sequencing library using the Illumina TruSeq Stranded mRNA Sample Preparation kit (Illumina) and RNA-seq was performed by the BMS Corporation according to the manufacturer′s protocol. Quality and size of libraries were assessed using an Agilent 2100 bioanalyzer DNA kit (Agilent). All libraries were quantified by qPCR using a CFX96 Real Time System (BioRad, Hercules, CA, USA) and sequenced on the NextSeq500 sequencers (Illumina) with a paired-end 75 bp plus single 8 bp index read run. To quantify the mapped reads on the reference genome into the gene expression values, Cufflinks [29] package with the strand-specific library option and other default options was used. The gene annotation of the reference genome hg19 from UCSC genome (https://genome.ucsc.edu) in GTF format was used as gene models and the expression values were calculated in fragments per kilobase of transcript per million fragments mapped (FPKM) unit. The differentially expressed genes (DEGs) between the two selected biological conditions were analyzed by Cuffdiff software in Cufflinks package [30] with the strand-specific library option and other default options. To compare the expression profiles of the samples, the normalized expression values of the thousands of DEGs that had been selected were unsupervised-clustered by in-house R scripts. In order to obtain GO term annotation results, the genes classified in RNA-Seq from g:Profiler were analyzed (https://biit.cs.ut.ee/gprofiler/gost).

### 2.5. Cell Survival/Proliferation and Alkaline Phosphatase (AP) Activity Analyses

For analyses of survival and proliferation, 5 × 10^4^ hiPSCs cells/well were seeded onto 12-well plates with and without the indicated flavonoids and incubated for 24 h. For cell survival and proliferation analyses, cells were treated with the indicated flavonoids for the indicated times and the cells were dissociated with StemPro Accutase and counted using a hemocytometer. All experiments were performed in triplicate. The AP activity was quantitated using the Alkaline Phosphatase Kit (Sigma) in accordance with the manufacturer′s protocol. Briefly, hiPSCs were fixed with citric acid–acetone formalin (Sigma) for 1 h at RT and then stained with naphthol/FRV-alkaline solution (Sigma) for AP staining. The AP activity was visualized with the phase contrast microscopy (FV-1000 spectral Olympus, Tokyo, Japan).

### 2.6. RNA Extraction and Quantitative Real-Time Reverse Transcriptase-Polymerase Chain Reaction (RT-PCR)

Total RNA was isolated from cells using Trizol reagent (iNtRON Biotechnology, Seongnam, Republic of Korea) according to the manufacturer′s instructions. For cDNA synthesis, 2 μg of total RNA was synthesized using 200 IU of M-MLV reverse transcriptase (Promega, Madison, WI, USA) in a 25 μL reaction containing 5× reaction buffer (50 mM Tris-HCl (pH 8.3 at 25 °C), 75 mM KCl, 3 mM MgCl_2_ and 10 mM DTT), 10 mM dNTP and 20 pmol Oligo (dT).

For PCR, 10 ng of cDNA template was used for each sample and subsequently mixed with 10 pmoles of forward and reverse primers, a 4 μL rTaq Plus 5× PCR Master Mix (ELPIS-BIOTECH, Daejeon, Republic of Korea), and added ddH2O up to 20 μL in total. Then, the PCR was performed using 9902 Veriti PCR Thermal Cycler Thermocycler PCR machine (ABI Applied Biosystems, Foster City, CA, USA). Additionally, PCR reactions were carried out under the following conditions: initial denaturing at 95 °C for 3 min and then 35 cycles of 10 s at 95 °C, 10 s at appropriate annealing temperature and 10 s at 72 °C, followed by 5 min at 72 °C. The PCR reactions were performed in triplicate. The products of PCR reaction were visualized in a 1.5–2% agarose gel (iNtRON) stained with ethidium bromide (Invitrogen, Carlsbad, CA, USA). The pictures of the visualized bands were captured under ultraviolet (UV) using a Canon Powershot A520 (Canon, Tokyo, Japan).

Real-time PCR was carried out using Applied Biosystems 7500 Real Time PCR System (Amersham Pharmacia Biotech, Little Chalfont, Bucks, U.K.) using Fast SYBR Green Master Mix (Applied Biosystems). The final volume of the PCR was 20 μL: 1 μL of cDNA sample, 10 μL of the SYBR Green Master Mix, 0.5 μL of each primer (10 pm), and 8 μL of ddH2O. PCR reactions were carried out under the following conditions: 95 °C for 30 s, followed by 40 cycles of 95 °C for 5 s and 60 °C for 10 s, and 72 °C for 10 s. The expression levels of the genes of interest were normalized to GAPDH. Fold change in gene expression was determined with the 2^−ΔΔCT^ method using GAPDH as the housekeeping gene [31].

### 2.7. Annexin V-PE Apoptosis Detection Analysis and Flow Cytometry 

hiPSC apoptosis detection analysis was performed using an Annexin V-PE apoptosis detection kit (BD Biosciences) following the manufacturer′s protocol. Briefly, the hiPSCs were subcultured with or without the indicated flavonoids and harvested after the indicated time. Cells were washed twice with cold PBS and then resuspended in 1× Binding Buffer at a concentration of 5 × 10^6^ cells/mL. Annexin V-PE and 7-aminoactinomycin D (7-AAD) were added to the cells with gentle vortexing and followed by incubation at RT (25 °C) for 20 min in the dark. Finally, 1× binding buffer was added to each tube and the flow cytometry analysis (BD FACSCalibur Flow Cytometer, BD Biosciences) was carried out within 1 h.

### 2.8. FreSHtracer Analysis for Detecting the Intracellular GSH Level of hiPSCs

hiPSCs were seeded at 1 × 10^4^ cells/well onto Matrigel-coated 96-well with bottom clear black plates (PerkinElmer, Waltham, MA, USA) in mTeSR1 medium. After 24 h, cells were exposed to the indicated amounts of flavonoids for 24 h at 37 °C and 5% CO_2_. After the end of the incubation period, media containing the indicated compounds was removed and cells were washed twice with 200 μL of HBSS/well (Hank′s Balanced Salt Solution, Thermo Fisher Scientific) using a multi-pipette (Thermo Fisher Scientific) to remove any remaining compounds. Then, 100 μL of 5 μM FreSHtracer from FreSHtracer Real-Time GSH Assay Kit (Cell2in, Seoul, Republic of Korea) was added per well and then incubated at 37 °C for 1 h. Afterwards, the FreSHtracer Real-Time GSH assay was carried out and the plate was treated with 3 mM of diamide for 5 min, then the supernatant was removed by suction. In order to obtain the fluorescence readings, the plate was inserted in the operetta (PerkinElmer) equipment and kept for 1 h. FreSHtracer analysis were performed in triplicate.

### 2.9. Fluorescence-Activated Cell Sorting (FACS) for GSH Level High Cells

To compare the quality of high GSH level and low GSH level cells, we tried to prepare the high GSH level cells and low GSH level cells using fluorescence-activated cell sorting (FACS) at F510 or F580, respectively, as follows. At first, 5 × 10^6^ hiPSCs were seeded and cultured in 100 mm cell culture dishes for three days with mTeSR1 medium which was changed every day. Then, the cells were treated with 5 μM FreSHtracer from FreSHtracer Real-Time GSH Assay Kit at 37 °C for 1 h. The FreSHtracer-treated cells were dissociated with Accutase and harvested using round-bottom tubes with a cell strainer cap (Corning, Corning, NY, USA). High GSH level or low GSH level cells were sorted using FACS at F510 or F580, respectively, and cultured in 60 mm cell culture dishes. Each experiment carried out in triplicate.

### 2.10. Western Blot Analysis

Protein extraction was employed using lysis buffer [1% Triton X-100 (Sigma), 100 mM Tris-HCl (pH 7.5), 10 mM NaCl, 10% glycerol (Amresco, Solon, OH, USA), 50 mM sodium fluoride (Sigma), 1 mM phenylmethylsulfonyl fluoride (PMSF; Sigma), 1 mM *p*-nitrophenyl phosphate (Sigma), and 1 mM sodium orthovanadate (Sigma)] and the cell lysates were centrifuged at 13,000 rpm for 15 min at 4 °C. The protein concentration in the supernatant was quantified using Bradford protein assay reagent (BioRad) and the proteins were separated with 8–12% sodium dodecyl sulfate polyacrylamide gel electrophoresis (SDS-PAGE). The separated proteins were then transferred onto nitrocellulose membranes (BioRad). After blocking with 5% skimmed milk in Tris-buffered saline for 1 h, the membranes were incubated with appropriate primary antibodies (Abs) against OCT4 (1:1000, Santa Cruz Biotechnology, Dallas, TX, USA), SOX2 (1:1000, Santa Cruz Biotechnology), NANOG (1:1000, Santa Cruz Biotechnology), or ACTIN (1:1000, Santa Cruz Biotechnology) overnight at 4 °C. Next, the membranes were incubated with the secondary Abs (anti-mouse, -goat, or -rabbit IgGs tagged with horse radish peroxidase (HRP)) for 1 h at 20 to 25 °C (Santa Cruz Biotechnology). Protein signals were visualized using an enhanced chemiluminescence (ECL) kit (DogenBio, Seoul, Republic of Korea). Each experiment carried out in triplicate.

### 2.11. Immunocy to Fluorescence Staining

For the immunofluorescence staining, hiPSCs were fixed in PBS containing 4% paraformaldehyde for 20 min at RT and then washed with PBS 3 times. The cells were permeabilized with 0.1% Triton X-100 in PBS for 10 min at RT and then subjected to blocking with 1% bovine serum albumin (BSA) (MP Biomedicals, Santa Ana, CA, USA) for 1 h at RT (25 °C). Afterwards, the cells were incubated with the primary Abs including stage-specific embryonic antigen (SSEA)-4 (1:100, Santa Cruz Biotechnology), tumor-related antigen (TRA)-1-60 (1:100, Santa Cruz Biotechnology), OCT4 (1:100, Santa Cruz Biotechnology), NANOG (1:100, Santa Cruz Biotechnology), or SOX2 (1:100, Santa Cruz Biotechnology) overnight at 4 °C and then washed 3 times with PBS. Following this process, the cells were incubated with the corresponding secondary Abs for 1 h at RT. Finally, hiPSCs were washed 3 times and stained with TOPRO3 (Invitrogen) for 10 min in order to visualize the nuclei. Fluorescent signals were examined using confocal microscope equipment (FV-1000 spectral).

### 2.12. Differentiation of hiPSCs into HPCs and NKCs

The hiPSCs were differentiated into HPCs using the embryoid bodied (EB)-based HPC differentiation method [32,33]. hiPSCs were briefly treated with the indicated flavonoids for 3 days before differentiation into EBs. For EB formation, cells were transferred onto Corning Ultra-Low Attachment Surface dishes with mTesR1 supplemented with 10 μM ROCK inhibitor for 6 days. On day 7, the formed EBs were transferred onto Matrigel-coated plate and incubated with the HPC differentiation medium (Iscove’s Modified Dulbecco′s Medium (Thermo Fisher Scientific) containing 20% FBS, with 100 ng/mL SCF (PeproTech), 10 ng/mL IL-3 (PeproTech), 10 ng/mL IL-6 (PeproTech), 20 ng/mL FLT3L (PeproTech), and 20 ng/mL BMP4 (PeproTech) with media exchange every 2 days until day 21. To obtain NKCs, hiPSC-derived HPCs were differentiated for 4 weeks in the presence of 10 ng/mL IL-15 (PeproTech), 5 ng/mL IL-3, 20 ng/mL IL-7 (PeproTech), 20 ng/mL SCF, and 10 ng/mL FLT3L. Medium containing cytokines was changed weekly with the exception of IL-3, which was only included for the first week of differentiation.

### 2.13. HSC (Hematopoietic Stem Cell) CFU (Colony-Forming Unit) Assay

CFU assays were performed in semi-solid medium supplemented with StemMACS HSC-CFU Media (MACS Miltenyi Biotec, Cologne, Germany) [34,35]. Briefly, cell suspension containing 1 × 10^5^ hiPSC-derived HPCs were mixed with 3 mL of StemMACS HSC-CFU Media (MACS Miltenyi Biotec, Bergisch Gladbach, NRW, Germany) and media mixture was subjected to a vortex until obtaining a homogenous mixture and then the tubes were incubated at RT for 10 min. The 1.1 mL aliquot of the mixture was then seeded using a sterile 5 mL syringe (KOVAX-SAFETY, Ansan, Republic of Korea) fitted with a 16-gauge blunt-end needle (STEMCELL Technologies) onto 35 mm Petri dishes. Finally, the 35 mm dishes were placed in a 100 mm dish. Another 35 mm dish containing 3 mL sterile water without lid was placed in 100 mm dish. All the dishes were incubated for 14–16 days in a humified incubator at 37 °C and 5% CO2 condition. The colonies were visualized by color and morphology with the phase contrast microscopy (FV-1000 spectral).

### 2.14. Statistical Analysis

Each experiment was repeated a minimum of three times. The data were presented as mean ± SEM. The statistical significance of differences between two groups was carried out using GraphPad Prism 7 software (GraphPad Software Inc., San Diego, CA, USA) and the *p* < 0.05 was considered significant.

## 3. Results

### 3.1. Generation and Characterization of PBMC-Derived hiPSCs (PBMC-hiPSCs)

**Figure 1:**
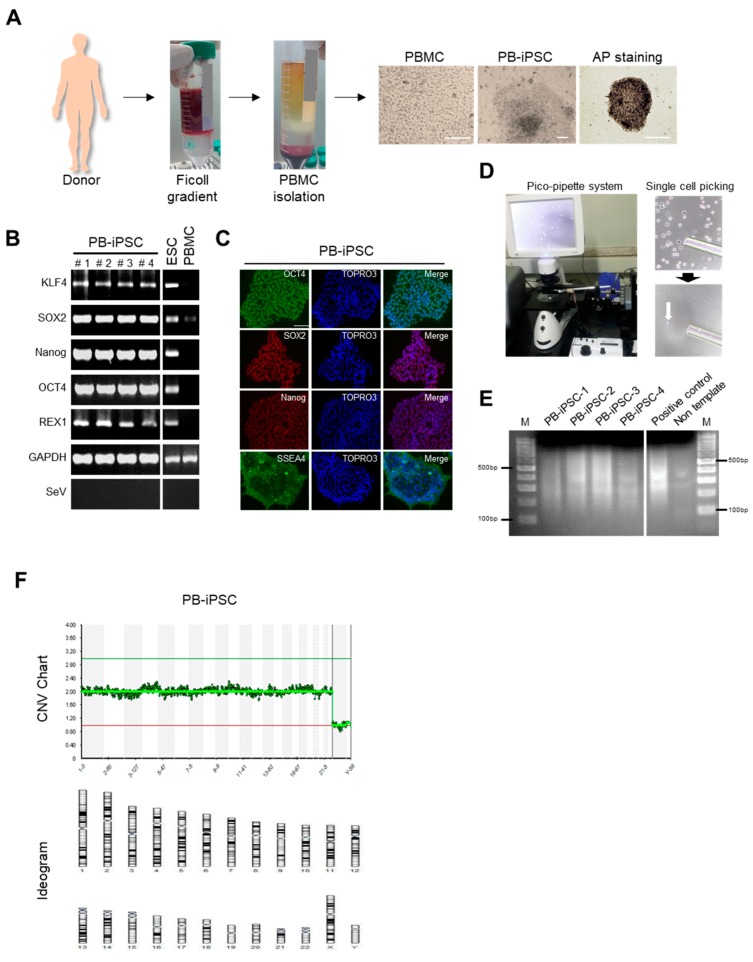
Reprogramming and characterization of hiPSCs. (**A**) Schematic representation of hiPSC generation from PBMCs. Phase contrast and AP staining photographs during hiPSC generation. Scale bar: 200 μm. Nikon Eclipse TE2000-U microscopy (Nikon Instruments Inc.). (**B**) RT-PCR showed that hiPSCs expressed endogenous KLF4, SOX2, NANOG and OCT4 genes, whereas exogenous reprogramming factors of the SeV were silenced. (**C**) Representative immunofluorescence analysis of hiPSCs showed the expression of human pluripotent stem cell-specific markers, such as NANOG, SOX2, OCT4 and SSEA4. Scale bar: 40 μm. (**D**) Images from the pico-pipette system controller and bright-field microscope used for picking up a single cell from a culture dish. (**E**) PCR detection of whole genome amplified single cell DNA samples. (**F**) Single-cell array-based comparative genomic hybridization (aCGH) sequencing for hiPSC chromosome abnormalities.

We generated PBMC-hiPSCs from healthy donor PBMCs and confirmed positive AP staining (Figure 1A). We also examined higher expression of reprogramming factors, such as Oct4, Sox2, Nanog, Rex1, and Klf4 in PBMC-hiPSCs than that in PBMCs, although there was weak expression of Sox2 gene in PBMCs, as reported previously [36,37,38,39] (Figure 1B). The expression of OCT4, SOX2, NANOG, and SSEA-4 in the representative iPSCs was also analyzed through immunostaining (Figure 1C).

**Figure 2:**
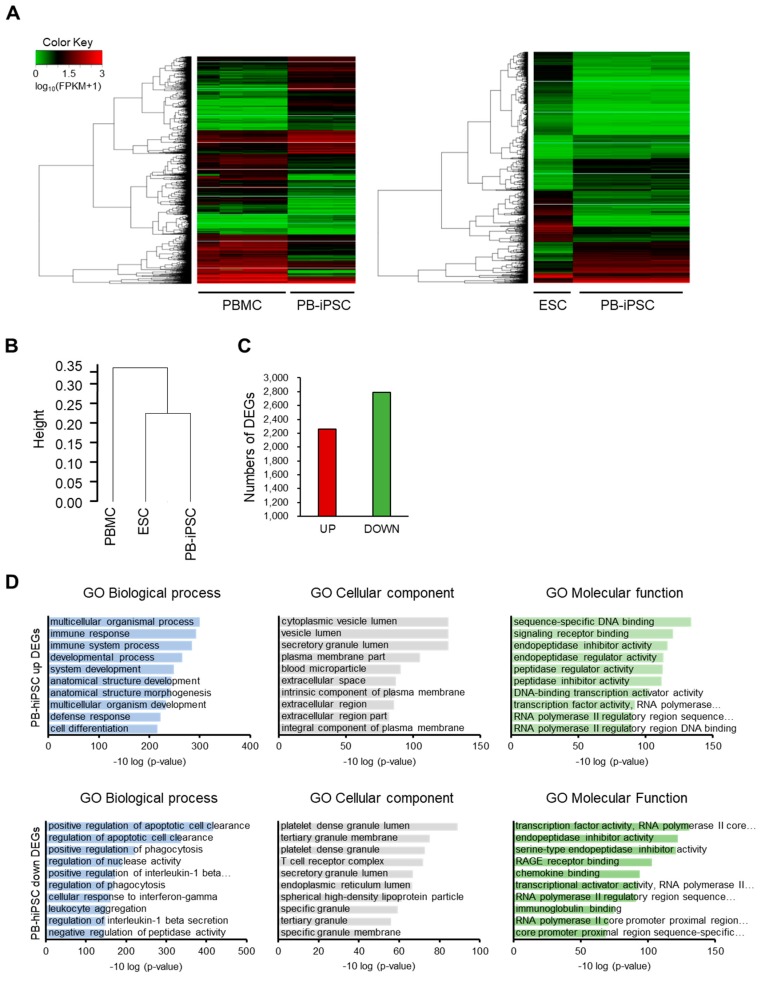
Global gene expression profiles of ESCs and PBMC-iPSCs, as revealed by RNA-sequencing. (**A**) Heatmap of hierarchical clustering of DEGs between ESCs and PBMC-iPSCs (Fold-change ≥ 2, *p*-value ≤ 0.05). (**B**) Dendrogram of hierarchical clustering analysis based on gene expression profiles to demonstrate the similarities between cells. (**C**) The bar graph shows the number of differentially expressed genes between ESCs and PBMC-iPSCs. (**D**) Functional enrichment analysis of highly regulated genes: ESCs vs PBMC-iPSCs and PBMC-iPSCs vs ESCs. Distribution of gene ontology (GO) terms of DEGs (Fold-change ≥ 2. *p*-value ≤ 0.05).

Next, 10 single cells per sample were randomly selected from the generated hiPSCs (Figure 1D) using a pico-pipette and WGA (Figure 1E) and NGS-based karyotype analysis of the single cell was conducted using the single cell NGS-based 24-chromosome aneuploidy screening protocol, which was performed by BMS Corporation [40,41]. The WGA and NGS-based karyotype analysis of the prepared single hiPSC cell revealed no chromosomal abnormality (Figure 1F and Supplementary Figure S1). Next, the expression profile of iPSCs was analyzed by RNA sequencing to examine variations in the expression of certain genes among PBMCs, PBMC-hiPSCs, and hESCs. We compared the DEG profiles of the PBMCs, the prepared hiPSCs, and the hESCs to analyze gene expression patterns (Figure 2). Heatmap of hierarchical clustering analysis showed differences in the transcriptional profiles of PBMCs, hiPSCs, and hESCs (Figure 2A). Hierarchical clustering analysis of the dendrograms of these gene expression profiles showed that gene expression of PBMC-iPSCs were more closely clustered to ESCs than PBMCs (Figure 2B). Although the generated hiPSCs showed similar gene expression patterns to those of ESCs, we found some differentially expressed genes (DEGs) between PBMC-hiPSCs and hESCs. We performed GO analysis to study the differences in gene function between ESCs and reprogrammed PB-iPSCs. We categorized 5048 DEGs in the GO analysis. Of these, about 2261 genes were upregulated (2 ≥ fold change, *p*-value ≤ 0.05) and 2787 genes were downregulated in PBMC-hiPSCs compared with their expression in ESCs (Figure 2C). The DEGs upregulated in PBMC-hiPSCs were included in three ontology categories (Biological Process, Cellular Component, and Molecular Function) and the top 10 were significantly enriched (2 ≥ fold change, *p*-value ≤ 0.05) within each ontology. When we compared the number of genes belonging to each category, the genes involved in multicellular organismal processes and immune responses were the most frequent in the “Biological Process” field (Figure 2D). In the “Cellular Component”, genes associated with the cytoplasmic vesicle lumen, the vesicle lumen, and the secretory granule lumen were the most highly distributed. In the “Molecular Function” section, genes involved in sequence-specific DNA binding, signaling receptor binding, and endopeptidase inhibitor activity were designated as the top three categories. In the GO analysis of the DEGs downregulated in PBMC-hiPSCs, the “Biological Process” included the top three categories, positive regulation of apoptotic cell clearance, and regulation of apoptotic cell clearance positive regulation of phagocytosis (Figure 2D). Platelet-dense granule lumen, tertiary granule membrane, and platelet-dense granule were enriched as the top three categories in the “Cellular Component” ontology. In the “Molecular Function” ontology, genes belonging to transcription factor activity, RNA polymerase II core promoter proximal region sequence-specific binding, endopeptidase inhibitor activity, and serine-type endopeptidase inhibitor activity were highly enriched.

### 3.2. 3,2′-DHF Treatment Promoted Proliferation and Suppressed Dissociation-Induced Apoptosis of hiPSCs

**Figure 3:**
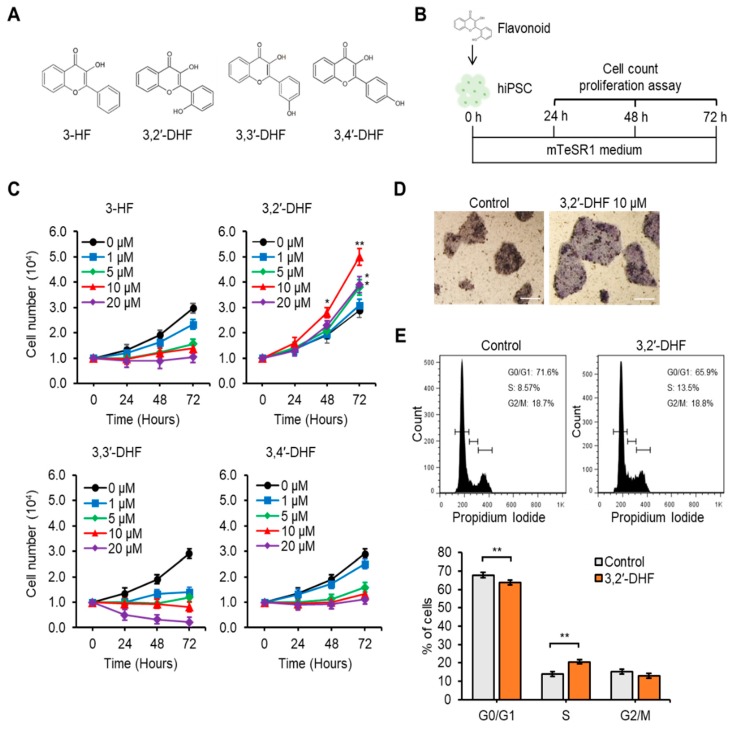
Effects of 3,2′-DHF on hiPSCs upon proliferation (**A**) Chemical structures of (3-HF), 3,2′-dihydroxyflavone (3,2′-DHF), 3,3′-DHF, and 3,4′-DHF. (**B**) Schematic representation of hiPSC proliferation with flavonoids. (**C**) Comparisons of the mean cell proliferation rate. 3-HF, 3,2′-DHF, 3,3′-DHF, 3,4′-DHF. (**D**) AP staining. The hiPSCs without (left panel) and with (right panel) 3,2′-DHF were photographed at 72 h. Scale bars: 200 μm. (**E**) Cell cycle analysis by flow cytometry using PI DNA staining of hiPSCs and 3,2′-DHF-treated hiPSCs. *n* = 3 biological samples. (* *p* < 0.05, ** *p* < 0.01).

Several kinds of flavonoids are known to regulate cell adhesion, proliferation, and differentiation in stem cells [42,43,44]. To determine the role of flavonoid of PBMC-hiPSCs, we added one of the flavonoids with differential structures during the culturing process of the hiPSCs (Supplementary Figure S2). Among the tested compounds, 3,2′-DHF significantly enhanced hiPSC proliferation (Figure 3). Although 3-HF, 3,2′-DHF, 3,3′-DHF, and 3,4′-DHF possess the same diphenylpropyl (C_6_C_3_C_6_) B ring of, the position of hydroxyl group around the B rings is different (Figure 3A) [24,45]. Treatment of the flavonoids resulted in the differences in hiPSC proliferation, the size and number of AP-positive colonies, and proliferation of PBMC-hiPSCs (Figure 3B–D). In particular, 3,2′-DHF treatment showed a significantly higher proliferation rate, higher number and bigger size of AP-positive colonies, and higher proliferation of PBMC-hiPSCs compared with other flavonoids. We also found that 3,2′-DHF treatment showed a significantly higher proliferation rate in other PBMC-iPSC lines (Supplementary Figure S3A). Next, we examined the effect of the concentration of the flavonoids on hiPSC proliferation. The trypan blue cell counting assay (Figure 3B,C) revealed that the number of hiPSCs was higher in groups treated with 10 μM 3,2′-DHF than in groups treated with other concentrations of 3,2′-DHF at 24 h, 48 h, and 72 h. On the other hand, hiPSCs treated with 3-HF, 3,3′-DHF and 3,4′-DHF showed a decreased proliferation rate (Figure 3C). After 72 h culture of hiPSC with or without 3,2′-DHF, we could detect higher number of AP-positive colonies in 3,2′-DHF-treated hiPSCs, compared with that in control cells (Figure 3D). We also found that hiPSCs treated with 3,2′-DHF showed higher S phase cells than did hiPSCs cultured without the flavonoid treatment (Figure 3E). In addition, we showed high cell survival rate and proliferation in the group treated with 10 μM of 3,2′-DHF even in ESC and several type of hiPSCs (U-iPSCs and DF-iPSCs) (Supplementary Figure S3B,C).

**Figure 4:**
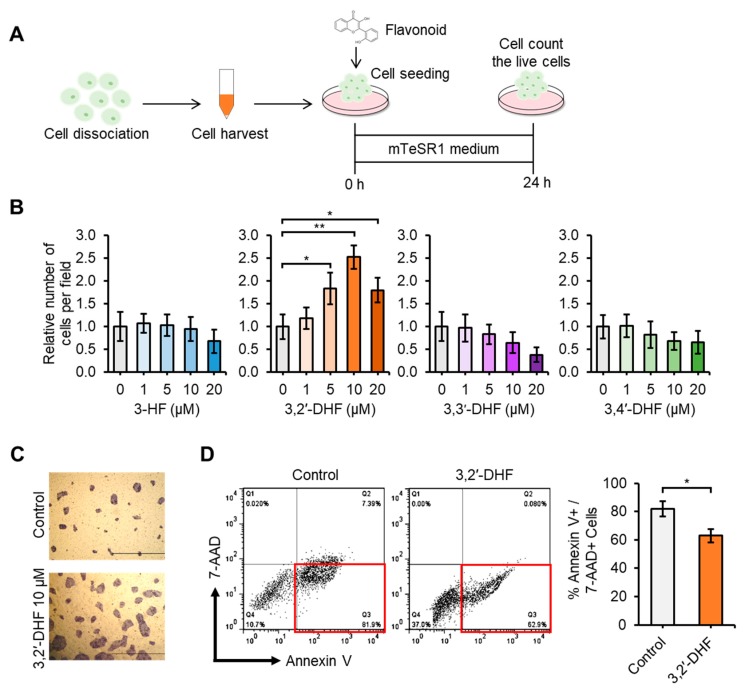
Effects of 3,2′-DHF on hiPSCs upon dissociation-induced apoptosis. (**A**) Schematic representation of hiPSC survival rate post dissociation-induced apoptosis upon subculture with flavonoids. (**B**) Survival rate of hiPSCs post dissociation-induced apoptosis upon subculture with Figure 5, 10 and 20 μM. 3-HF, 3,2′-DHF, 3,3′-DHF, 3,4′-DHF. Comparisons of the relative number of cells per field. Scale bars: 500 μm. (**C**) hiPSC colonies are shown after cell seeding for 24 h and staining with alkaline phosphatase (AP). (**D**) Apoptotic effects of control hiPSCs and 3,2′-DHF-treated hiPSCs upon flow cytometry analysis of annexin V+ and 7-AAD- cells. *n* = 3 biological samples. (* *p* < 0.05, ** *p* < 0.01).

The suppressive effect of flavonoids on the dissociation-induced apoptosis was measured after flavonoids were treated at various concentration (Figure 4A–D). There was no statistically significant change in dissociation-induced apoptosis rate in 3-HF, 3,3′-DHF and 3,4′-DHF-treated hiPSCs. The suppression of the dissociation-induced apoptosis rate was significantly increased in hiPSCs treated with 1 μM, 5 μM, 10 μM, and 20 μM 3,2′-DHF compared with that of the control group (Figure 4B). The suppression rate increased most significantly when hiPSCs were treated with 10 μM 3,2′-DHF. In addition, we detected high numbers of AP staining-positive cells in the 10 μM 3,2′-DHF-treated cells (Figure 4C). We then measured the dissociation-induced apoptosis in hiPSCs by Annexin V/7-AAD staining [46]. The hiPSCs were dissociated with Accutase and Annexin V +/ 7-AAD + cells were measured after 12 h. The 3,2′-DHF treatment led to a significant decrease in the Annexin V+/ 7-AAD- cell population (Figure 4D).

Next, although human ESCs and iPSCs are usually in a so-called “primed” pluripotent state that resembles the post-implantation epiblast [47], we tried to culture the naïve state hiPSCs. Compared with the primed state hiPSCs, naïve state hiPSCs are characterized by a higher growth rate, more efficient clonal growth from single cells, and a higher propensity to differentiate to another lineage [47,48,49]. Previously, we reported that treatment with 3,2′-DHF enhanced the proliferation rate, pluripotency marker expression, and neuroprotective properties of naïve state mPSCs [24]. Here, we examined the effect of 3,2′-DHF treatment on naïve state hiPSCs. First, we prepared the naïve state hiPSCs by culturing the primed hiPSCs in mTeSR1 + 2iFL + LPA medium [28] (Supplementary Figure S4A). We detected the high mRNA and protein expression levels of YAP in LPA-treated hiPSC compared with the untreated group (Supplementary Figure S4B,C). In particular, higher expression of the naïve state PSCs-specific genes, including inhibitor of DNA binding 3 (ID3), zinc family member 1 (ZIC1), and transcription factor binding to IGHM enhancer 3 (TFE3), was observed in the naïve state hiPSCs compared with that in the primed hiPSCs. The expressions of the primed state-associated genes, mix paired-like homeobox (MIXL1) and eomesodermin (EOMES) [50], was increased in the primed hiPSCs than in the naïve state hiPSCs (Supplementary Figure S4D). We found that as hiPSCs were cultured, the primed state hiPSCs, which exhibit a flat colony morphology, were transformed to a dome-like morphology (Supplementary Figure S4E), which is specific to naïve state hiPSCs [28]. In the immunocytochemistry staining, the naïve state hiPSCs found to express high levels of pluripotency markers, such as OCT4, Tra-1-60, Tra-1-81, and SSEA4 (Supplementary Figure S4F). Furthermore, the LPA-induced expression of YAP was confirmed to be increased in the naïve state hiPSCs. Importantly, treatment with 3,2′-DHF led to a significant increase in cell proliferation (Supplementary Figure S4G), suppression of dissociation-induced apoptosis (Supplementary Figure S4H), and enhanced expression of naïve state-specific markers and suppressed expression of MIXL1, a primed state-associated gene (Supplementary Figure S4I). Treatment with 3,2′-DHF did not show any observable effect on the dome-like morphology of the naïve state hiPSCs (Supplementary Figure S4J).

### 3.3. Additional Treatment with 3,2′-DHF and Y-27632 Led to Substantial Improvement of Cell Proliferation and Adhesion

**Figure 5:**
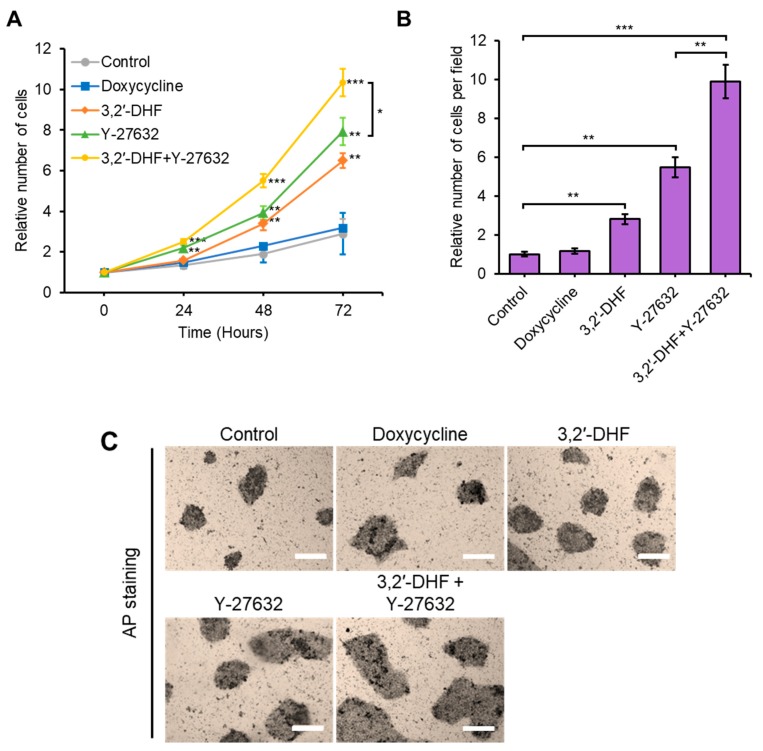
Effect of 3,2′-DHF, Y-27632 and doxycycline on hiPSCs upon proliferation and dissociation-induced apoptosis. (**A**) Comparisons of the mean cell proliferation rate upon treatment with Doxycycline (1 μg/mL), 3,2′-DHF (10 μM), and Y-27632 (10 μM). (**B**) Effects of doxycycline, 3,2′-DHF, and Y-27632 on hiPSCs upon dissociation-induced apoptosis. Comparisons of the mean number of cells per field. (**C**) AP staining. Cells treated with doxycycline, 3,2′-DHF and Y-27632. Scale bars: 200 μm. *n* = 3 biological samples. (* *p* < 0.05, ** *p* < 0.01, *** *p* < 0.001).

As previous reports suggested, supplementation with doxycycline [51] or the ROCK inhibitor, Y-27632 [52], could increase the cell proliferation rate and cell survival of hiPSCs. We tried to examine the effect of additional treatment with 3,2′-DHF, doxycycline, or Y-27632. Dissociated hiPSCs were plated on Matrigel-coated plates and treated with each chemical. Treatment with doxycycline (1 μg/mL) alone showed no significant improvement in cell survival upon dissociation-induced apoptosis and proliferation (Figure 5A,B). Importantly, co-treatment with 3,2′-DHF and Y-27632 led to substantial improvements in cell proliferation and suppression of dissociation-induced apoptosis of hiPSCs (Figure 5A,B). Co-treatment with 3,2′-DHF and Y-27632 also resulted in significant improvements in both the number and morphology of AP-positive colonies (Figure 5C), suggesting that 3,2′-DHF and Y-27632 have differential and synergistic actions in improving hiPSC adhesion and proliferation.

**Figure 6:**
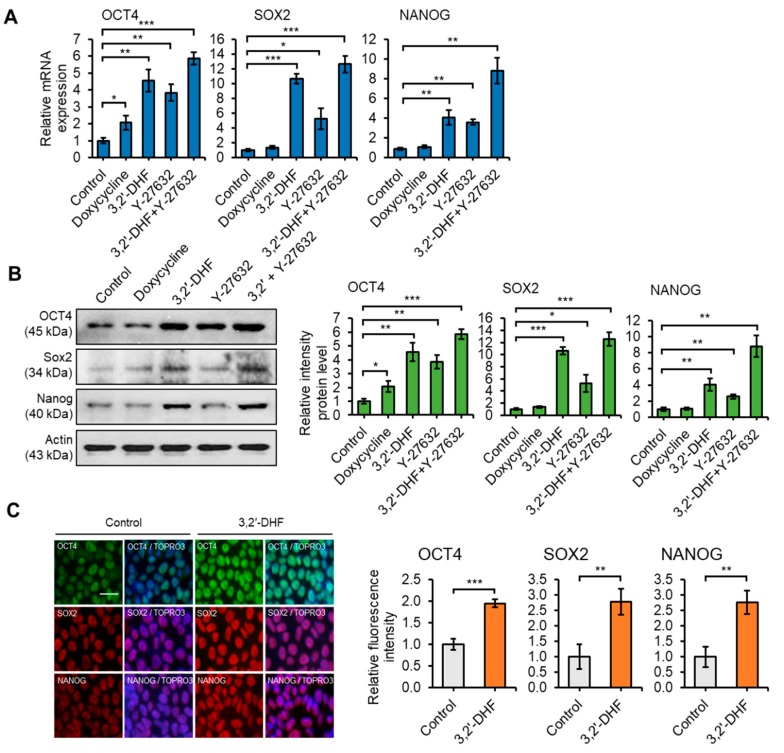
3,2′-DHF regulates hiPSC pluripotency. (**A**) Relative mRNA expression of 3,2′-DHF was higher in hiPSCs. (**B**) Western blot analysis and relative signal intensity of OCT4, SOX2, and NANOG proteins in hiPSCs. (**C**) Immunofluorescence staining for OCT4, SOX2, and NANOG in hiPSCs cultured with or without 3,2′-DHF and relative fluorescence intensity. Images were obtained at the same magnifications. Scale bars: 10 μm. *n* = 3 biological samples. (* *p* < 0.05, ** *p* < 0.01, *** *p* < 0.001).

It is well known that Oct4, Sox2 and Nanog play essential roles in maintaining the undifferentiated state of ESCs and also regulate cell growth and apoptosis [53,54]. In addition, Oct4 downregulation in human and mouse ESCs inhibits cell proliferation by cell cycle arrest in the G0/G1 phase, and the overall integrity of the Oct-4 functional domains is important for the activation of S-phase entry [55]. We subsequently compared mRNA and protein levels of the pluripotency markers, Oct4, Sox2, and Nanog, in hiPSCs with and without 3,2′-DHF and Y-27632 treatment (Figure 6A–C). Quantitative RT-PCR and western blot analysis showed that mRNA and protein levels of Oct4, Sox2 and Nanog were significantly increased following treatment with 3,2′-DHF alone or co-treatment with 3,2′-DHF and Y-27632 (Figure 6A,B). This result suggested that 3,2′-DHF and Y-27632 may have differential and synergistic effects on the proliferation and pluripotency markers expression of hiPSCs. Immunocytochemistry data also confirmed that 3,2′-DHF treatment led enhanced the expression of pluripotency markers (Figure 6C).

### 3.4. Treatment with 3,2′-DHF Increased the Ratio of GSH High Cells

**Figure 7:**
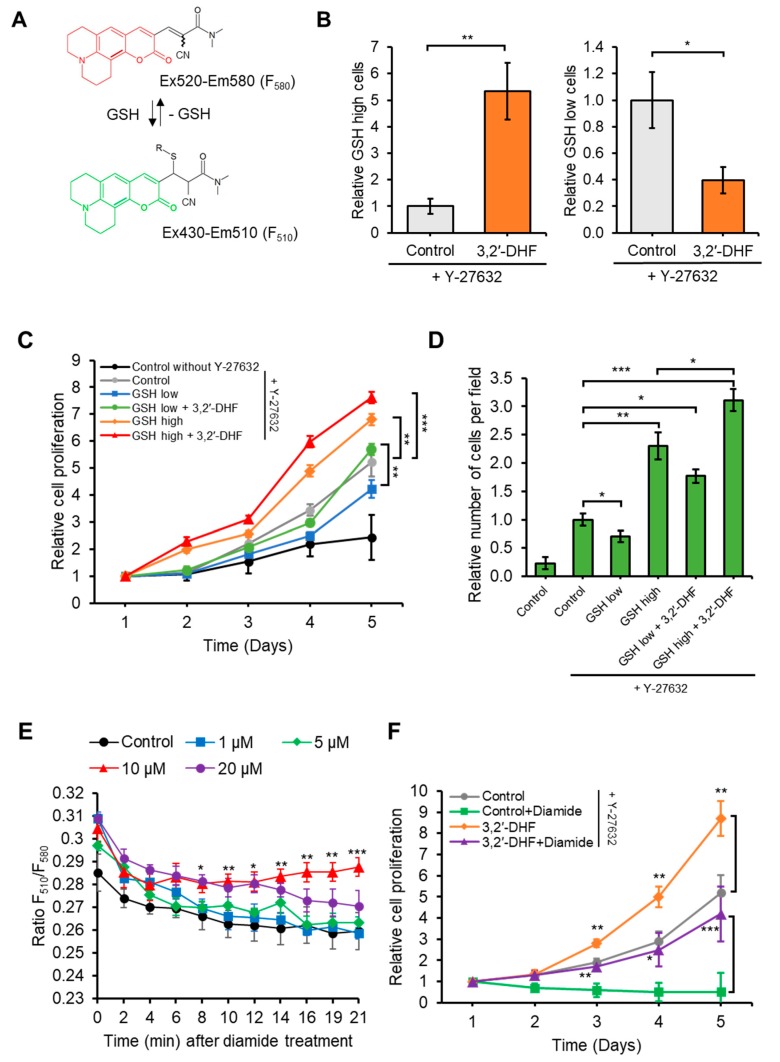
3,2′-DHF regulates GSH levels. (**A**) Changes in the absorption and fluorescence spectra of FreSHtracer when reacted with increasing concentrations of glutathione (GSH). (**B**) Relative number of GSH-high and -low hiPSCs after treatment of 3,2′-DHF and/or Y-27632. (**C**) Effects of low and high concentrations of GSH on hiPSCs upon dissociation-induced apoptosis. (**D**) Effects of low and high concentrations of GSH on hiPSCs upon proliferation for 5 days. (**E**) Effect of 3,2′-DHF on the GSH-specific reaction of FreSHtracer with diamide. (**F**) Comparisons of the mean cell proliferation rate with. 3,2′-DHF and diamide treatment. *n* = 3 biological samples. (* *p* < 0.05, ** *p* < 0.01, *** *p* < 0.001).

To assess the effect of 3,2′-DHF treatment on the quality of PBMC-iPSCs, the real-time measurement of GSH level was performed using a FreSHtracer kit, a newly developed reversible fluorescent dye for measuring the level GSH in living cells [56,57]. A recent study showed that when FreSHtracer is reversibly bound to GSH, it fluoresces at a peak of 510 nm (F510, λex 430 nm: green fluorescence) and when it is released from GSH, it fluoresces at 580 nm (F580, λex 520 nm: red fluorescence) (Figure 7A). We found that the percentage of GSH-high cells (F510) in the 3,2′-DHF-treated hiPSC increased significantly (about 5-fold) than those in the control cells without 3,2′-DHF treatment. In addition, the percentage of GSH-low (F580) cells in the 3,2′-DHF-treated hiPSC was significantly decreased compared to control cells. Therefore, treatment of 3,2′-DHF led to conversion of GSH-low cells and GSH-mid cells into the GSH-high cells (Figure 7B). Next, we prepared the GSH-high or GSH-low cells by FACS and measured the proliferation (Figure 7C) and cell survival upon dissociation-induced apoptosis rates (Figure 7D) of the GSH-high or GSH-low cells. Compared with the control cells (the unsorted PBMC-hiPSCs containing both the GSH-high and GSH-low cells without 3,2′-DHF treatment), GSH-low PBMC-hiPSCs showed lower proliferation and survival rates, whereas GSH-high cells showed enhanced proliferation and survival upon dissociation-induced apoptosis (Figure 7C,D). When either GSH-low or GSH-high cells were treated with 3,2′-DHF, the proliferation and dissociation-induced apoptosis rates of the GSH-low + 3,2′-DHF or GSH-high + 3,2′-DHF were significantly increased, indicating that treatment with 3,2′-DHF enhanced the proliferation and increased the survival rate following dissociation-induced apoptosis.

We then assessed the GSH level of 3,2′-DHF-treated PBMC-hiPSCs by measuring the G-S-S-G reducing capacity of the live cells. Cells were pretreated with different doses (0, 1, 5, 10, and 20 μM) of 3,2′-DHF and then the 3,2′-DHF-treated PBMC-hiPSCs were treated with diamide, thiol-specific oxidants [58] and the GSH levels were measured for 21 min using a FreSHtracer kit. Treatment with diamide led to a marked decrease in GSH level, but, compared with the control cells (3,2′-DHFuntreated cells), the 3,2′-DHF-treated PBMC-hiPSCs showed more rapid recovery from the diamide-induced oxidation of GSH groups. This result demonstrated the higher G-S-S-G reducing capacity of the 3,2′-DHF-treated cells (Figure 7E). As the PBMC-hiPSCs were treated with 10 μM 3,2′-DHF, the diamide-induced oxidation was recovered rapidly (Figure 7E) and dramatic increases were seen in long-term (5 day) survival and proliferation, even after diamide treatment (Figure 7F). After diamide treatment, the PBMC-hiPSCs cultured without 3,2′-DHF could not survive and showed almost no increase in cell numbers for 5 days of culturing (Figure 7F). We also confirmed that 3,2′-DHF and Y-27632 have differential and synergistic effects on GSH regeneration or the G-S-S-G reducing capacity of hiPSCs. Taken together, our results reveal that 3,2′-DHF treatment enhances the GSH level of hiPSCs by increasing the GSH regenerating or G-S-S-G reducing capacity.

### 3.5. 3,2′-DHF Regulates the Differentiation Potential and HPC Differentiation of PBMC-hiPSCs

**Figure 8:**
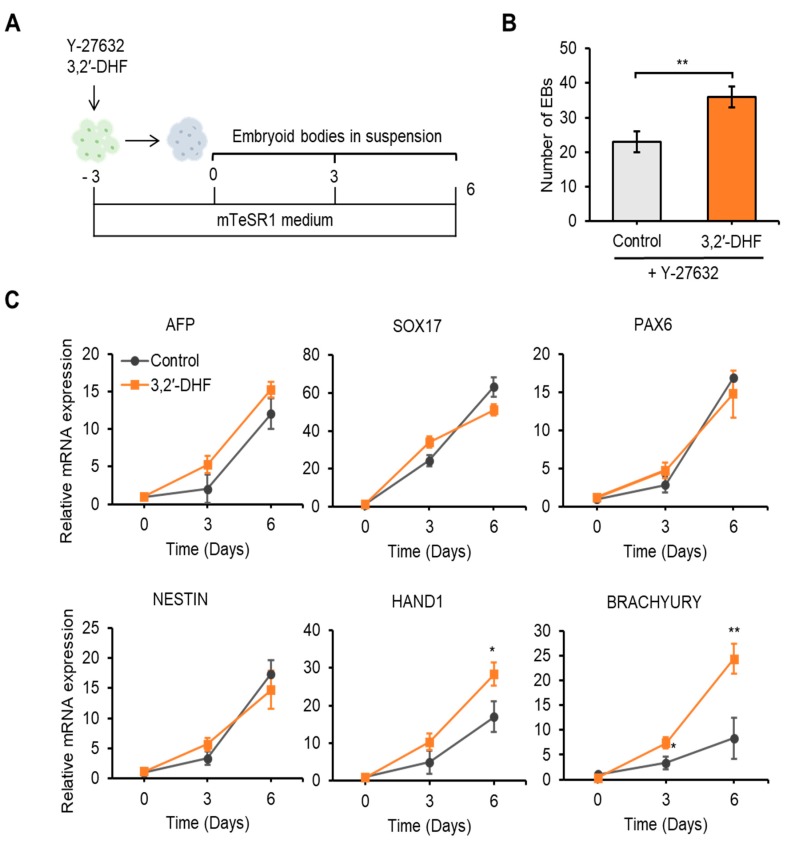
Germ layer-specific gene expression. (**A**) Scheme of EB formation process. (**B**) Number of EBs with or without 3,2′-DHF after 6 days. (**C**) Human PSC cultures treated with or without 3,2′-DHF were analyzed at days 0, 3, and 6 during EB formation by qRT-PCR for developmental genes, including BRACHYURY (mesoderm), HAND1 (mesoderm), SOX17 (endoderm), AFP (endoderm). PAX6 (ectoderm), and NESTIN (ectoderm). *n* = 3 biological samples. (* *p* < 0.05, ** *p* < 0.01).

Flavonoids are known to regulate stem cell differentiation, such as osteogenic differentiation [42], adipogenic differentiation [23], and neuronal differentiation [43]. We investigated the effect of 3,2′-DHF on the differentiation of hiPSCs. Three days prior to the induction of spontaneous differentiation, the cells were treated with 3,2′-DHF. On day 4, the generated EBs were transferred to mTeSR1 medium and cultured for 6 days to monitor spontaneous differentiation. Cells were harvested and tested for the expression of endoderm, mesoderm, and ectoderm markers on days 3 and 6 during EB formation (Figure 8A). It is worth noting that 3,2′-DHF-treated groups showed more EB production (Figure 8B). We assessed the expression of endoderm markers, such as α-fetoprotein (AFP) and SRY-related HMG-box 17 (SOX17); ectoderm markers, such as paired box 6 (PAX6) and NESTIN; and mesoderm markers, such as heart and neural crest-derived transcript-1 (HAND1) and BRACHYURY. The mRNA expression of the three germ markers was examined by qPCR on day 0 before EB formation and on days 3 and 6 during EB formation, and the mRNA levels of the mesoderm markers HAND1 and BRACHYURY were increased in the 3,2′-DHF-treated EBs compared with that in the controls (Figure 8C).

**Figure 9:**
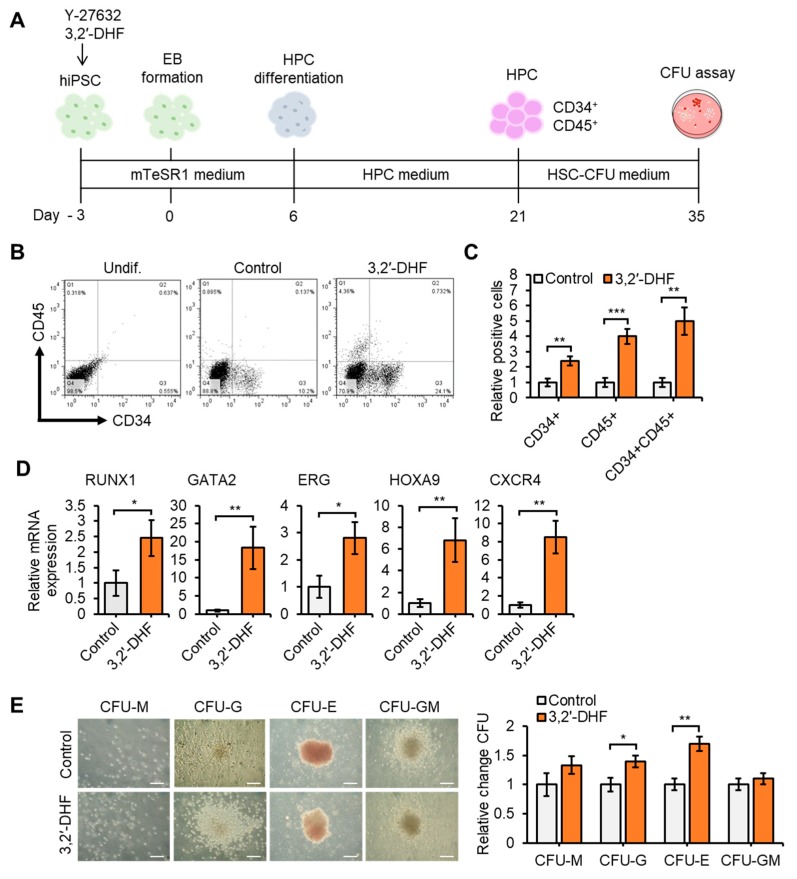

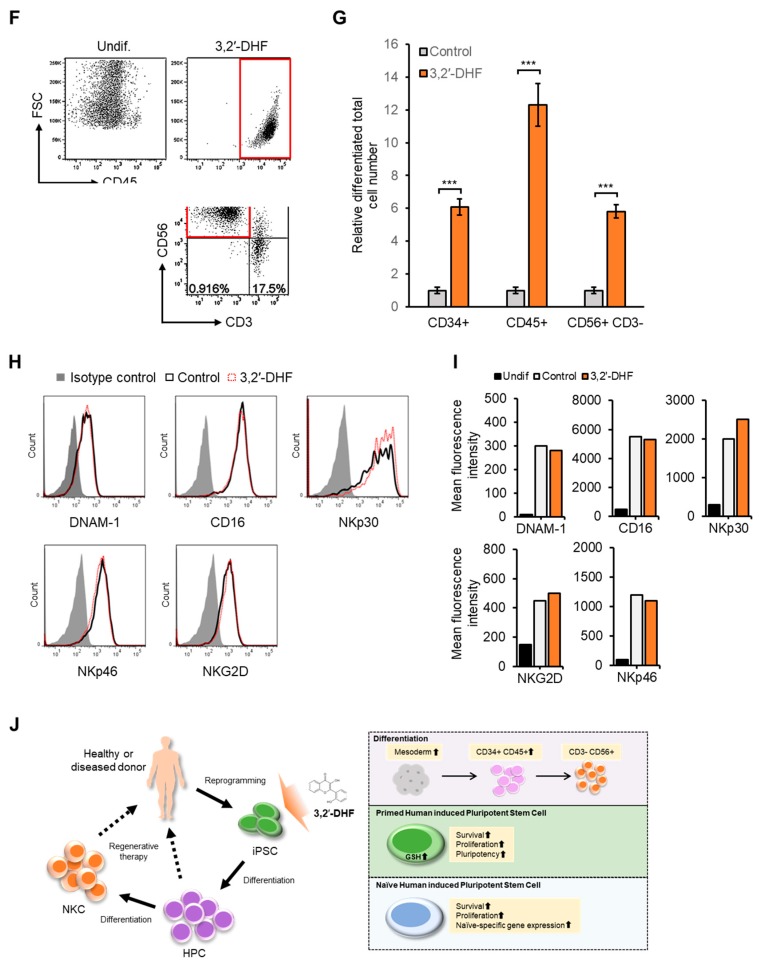
3,2′-DHF regulates HPC differentiation. (**A**) Scheme of HPC differentiation process. (**B**) Representative flow cytometry dot plots of hematopoietic markers during hiPSC differentiation of control or 3,2′-DHF-treated cells. (**C**) Percentage of CD34+ and CD45+ cells during hematopoietic differentiation. (**D**) mRNA expression of HSC markers. (**E**) Colony forming unit (CFU) potential of control and 3,2′-DHF-treated hiPSCs. Fold change in CFUs with control or 3,2′-DHF-treated cells. Scale bar: 100 μm. (**F**) hiPSC-HPC-derived NK differentiation with or without 3,2′-DHF. (**G**) Total number of cells differentiated into CD34+ and CD56+CD3- cells from hiPSCs. (**H**) NK marker populations in hiPSC-HPC-derived NK cells. (**I**) Mean fluorescence intensity of NK markers in hiPSC-HPC-derived NK cells. (**J**) Schematic diagram illustrating the modulation of hiPSCs function after 3,2′-DHF-treatment with the further therapeutic application. Biological replicates (*n* = 3) are represented in C, D, E and G. (* *p* < 0.05, ** *p* < 0.01, *** *p* < 0.001).

To study the detailed effect of 3,2′-DHF treatment on mesodermal specification, we developed a 3-stage procedure to direct the differentiation of hiPSCs into the hematopoietic lineage. After 3 days of treatment with 3,2′-DHF, the hiPSCs were cultured for 3 days and EBs were formed on the 6th day. Then, the EBs were cultured in an HPC medium for 15 days (Supplementary Figure S5). After HPC differentiation, HPC markers such as CD34+ and CD45+ were analyzed by flow cytometric analysis. HPCs differentiated from hiPSCs were also tested for their potential to impact other blood cells using a CFU assay (Figure 9A). We found that the hiPSCs successfully differentiated into HPCs (Figure 9B), and the group treated with 3,2′-DHF showed significantly increased populations of CD34+ CD45+ cells (Figure 9C). Cell identity of HPCs can be defined by gene regulation networks governed by transcription [59] and several previous studies have shown that various transcription factors regulate HPC differentiation and engraftment [60,61,62]. We investigated whether 3,2′-DHF regulates transcription factors after hiPSC differentiation into HPCs and confirmed that the expression of RUNX1, GATA2, ERG, HOXA9, and CXCR4 were increased in the 3,2′-DHF-treated cells (Figure 9D). Moreover, HPCs derived from the 3,2′-DHF-treated cells showed more CFU capacity, especially CFU-G and CFU-E, than the controls (Figure 9E).

NK cells are a type of CD3−CD56+ lymphoid cells that are essential for the innate immune system, and their broad cytotoxicity and rapid killing capacity make them ideal for use in cancer immunotherapy [63]. In addition, several clinical trials on CAR-NK cells have been carried out during 2016 and 2018 [64]. Here, we attempted to differentiate hiPSC-HPCs into NK cells (Figure 9F–I) and found that 3,2′-DHF-treated cells successfully differentiated into cells expressing the NK cells surface marker CD45+ (upper red rectangle) and CD56+ CD3− (lower red rectangle) (Figure 9F). Overall, 3,2′-DHF treatment led to approximately 6-fold CD34+, 12-fold CD45+ and 5.8-fold increases in the production of HPCs and NK cells, respectively (Figure 9G). We also detected significant expression of the NK cell markers, DNAM-1, CD16, NKG2D, and NKp46 (Figure 9H–I).

## 4. Discussion

Flavonoids are subdivided into flavonol, flavone, flavanone, flavanol, isoflavone, and anthocyanidin groups, in which two benzene rings (C_6_) are connected via three carbon atoms (C_3_) [65]. Flavonoids are known to regulate cell adhesion, proliferation, and differentiation in stem cells [42,43,44]. To determine the role of flavonoid of hiPSCs, we screened 39 flavonoids and found that treatment with 3,2′-DHF led to significant increases in cell proliferation and expression of naïve state stemness markers and decreases in dissociation-induced apoptosis of hiPSCs (Supplementary Figure S4). We found that 3,2′-DHF affects proliferation and differentiation in both mouse and human PSCs [24]. Our data showed that 3,2′-DHF works synergistically with a ROCK inhibitor, Y-27632, in enhancing survival of cells from dissociation-induced apoptosis and cell proliferation (Figure 5 and Figure 6). Therefore, we selected 3,2′-DHF based on its potential to promote the cell proliferation not based on its antioxidant activity.

We also explored the survival of the cells from dissociation-induced apoptosis and cell proliferation of naïve state hiPSCs treated with 3,2′-DHF. When cultured in mTeSR1 + 2iFL + LPA medium, primed hiPSCs are converted into naïve hiPSCs and induce the YAP signaling pathway [28]. Importantly, we found that 3,2′-DHF treatment resulted in an increased cell survival rate and proliferation in naïve state hiPSCs (Supplementary Figure S4). We also examined if 3,2′-DHF treatment led to increase in the expression of hiPSC naïve specific markers.

Reactive oxygen species (ROS)-mediated oxidation plays an important role in regulating various signaling proteins that affect self-renewal, pluripotency, viability, and genomic stability of stem cells [66]. Cellular redox homeostasis depends on the balance between ROS production and elimination by several enzymes and antioxidants, such as GSH [67,68]. Owing to its high intracellular concentrations, GSH, the thiol-containing tripeptides, plays the most important role in maintaining cellular redox homeostasis [69]. The protective effect of flavonoids in biological systems may be due to electron-free radical transport, metal catalyzed chelation, antioxidant enzyme activation, α-tocopherol radical reduction, and oxidant inhibition [70]. In addition, various studies have shown that the antioxidant flavonoids, such as rutin and quercetin, could induce glutathione production and glutathione peroxidase activation [71,72,73]. Here, we used a real-time GSH assay system, FreSHtracer, to reversibly measure the intracellular levels of GSH in hiPSCs [56]. We found that more GSH-high cells were detected in the group treated with 3,2′-DHF, and that these GSH-high cells showed increased hiPSC survival and proliferation (Figure 7). Notably, treatment with 3,2′-DHF in GSH-high cells showed a significant increase in cell survival and proliferation, indicating that 3,2′-DHF can modulate GSH levels. High GSH levels are essential for protection from DNA damage in hiPSC [74], and for regulation of the pluripotency-related transcription factor OCT4 [75]. We found that 3,2′-DHF treatment also increased the level of pluripotency marker in hiPSC; especially, 3,2′-DHF treatment led to increased NANOG expression, which may be important in regulating S-phase entry in stem cells (Figure 5). Our study confirmed that, when treated with 3,2′-DHF, more hiPSCs were in S phase compared to the hiPSCs cultured without the flavonoid (Figure 3E). A previous study reported that overexpression of NANOG considerably increased proliferation by binding to regulatory regions of CDK6 and CDC25A, two crucial cell cycle regulators [76].

Ontology analysis of DEGs revealed that PBMC-iPSCs have enriched gene expression related with immune response and immune system process compared to the ESCs, which may reflect the maintenance of epigenetic memory after the reprogramming processes [77]. We showed higher efficiency of HPC differentiation in PBMC-iPSC than that in U-iPSC (data not shown). Combined with these results, it may be meaningful to differentiate into HPC or NK cells using PBMC-iPSC. Additionally, to gain insight into whether 3,2′-DHF plays a role in promoting hiPSC differentiation, we examined expressions of three germ layer markers—endoderm (AFP and SOX17), ectoderm (PAX6 and NESTIN), and mesoderm (HAND1 and BRACHYURY)—during differentiation within EBs. Importantly, we found that treatment with 3,2′-DHF led to a significant upregulation in HAND1 and BRACHYURY expression during spontaneous differentiation toward the three germ lineages within EBs (Figure 8). This result is different from what has been observed for mPSCs. In mPSCs, expression of all three germ layer makers were upregulated upon 3,2′-DHF treatment while mesoderm markers were dominantly increased in hPSCs upon same treatment. As hESCs and mPSCs represent different stages of pluripotency, our study suggests that they may also have distinct responses to the 3,2′-DHF treatment. As hESCs and mPSCs represent different stages of pluripotency, our study suggests that they may also have distinct responses to the 3,2′-DHF treatment. Examples where hiPSCs and mPSCs respond differently to similar transcriptional and signaling pathways have been reported [48]. Our results extend these findings by showing that 3,2′-DHF is involved in the mesodermal differentiation of hiPSCs. Next, we observed that differentiation into HPCs (CD34+ CD45+) and their CFU potential were significantly increased by 3,2′-DHF pretreatments (Figure 9). We observed that CD45+ cells showed about 5 times higher population with 3,2′-DHF pretreatments in flow cytometry experiments after HPC differentiation. This suggests that 3,2′-DHF might push the hematopoietic differentiation towards the lymphoid lineage [78]. To confirm this, we can extend the gene expression study on the genes specific to the different hematopoietic lineages.

## 5. Conclusions

Taken together, we found that cell proliferation increased during the HPC differentiation and, as a result of this, 3,2′-DHF pretreatments led to an approximately 6-fold increase in the production of HPCs and NK cells.

## Supplementary Materials

The following are available online at https://www.mdpi.com/2077-0383/9/3/669/s1, Figure S1: Chromosome abnormalities of PBMC-hiPSCs, Figure S2: List of names, subclasses, and structures of tested flavonoids with their effect on stem cell proliferation, Figure S3: (A) Proliferation effects of 10 μM 3,2′-DHF treatment on PB-iPSC lines. (B) Survival upon dissociation-induced apoptosis condition on several types of hPSCs. (C) Proliferation effect of 10 μM 3,2′-DHF treatment on several types of hPSCs, Figure S4: Derivation of naïve state hiPSCs, Figure S5: hiPSC-derived HPC differentiation morphology at days 8, 12, 14 and 16 with or without 3,2′-DHF, Table S1: Primer sequences.
