# Supplementary material for: 3,2′-Dihydroxyflavone Improves the Proliferation and Survival of Human Pluripotent Stem Cells and Their Differentiation into Hematopoietic Progenitor Cells
Source: J Clin Med. 2020 Mar 2;9(3):669. doi: 10.3390/jcm9030669 (PMC7141312; doi:10.3390/jcm9030669)
Supplement: Supplementary file 1 [file jcm-09-00669-s001.pdf]

Journal of
Clinical Medicine

Supplementary Table 1. Primer sequences

Accession no.	Gene	Forward primer sequence (5′-3′)	Reverse primer sequence (5′-3′)	Application
NM_004235.4	KLF4	ACCTACACAAAGAGTTCCCATC	TGTGTTTACGGTAGTGCCTG	undifferentiated hiPSC markers
NM_002701.5	OCT4	CAATTTGCCAAGCTCCTGAAG	GTTGCCTCTCACTCGGTTC	
NM_003106.3	SOX2	TTCACATGTCCCAGCACTAC	TCCATGCTGTTTCTTACTCTC	
NM_024865.3	NANOG	CAGAAATACCTCAGCCTCCAG	GCCACCTCTTAGATTTCATTCTCTG	
NM_174900	REX1	CAGATCCTAAACAGCTCGCAGAAT	GCGTACGCAAATTAAAGTCCAGA	
NM_002046.5	GAPDH	AATCCCATCACCATCTTCCAG	ATGACCCTTTTGGCTCCC	housekeeping gene
	SeV	GGATCACTAGGTGATATCGAGC	ACCAGACAAGAGTTTAAGAGATA TGTATC	Sendai virus transgene-specific primer
NM_001134	AFP	CTGCAATTGAGAAACCCACTG	TTCCCTCTTCACTTTGGCTG	endoderm markers
NM_022454	SOX17	AGAATCCAGACCTGCACAAC	GCCGGTACTTGTAGTTGGG	
NM_000280	PAX6	GCCCTCACAAACACCTACAG	TCATAACTCCGCCCATTCAC	ectoderm markers
NM_006617	NESTIN	TGCGGGCTACTGAAAAGTTC	GGCTGAGGGACATCTTGAG	
NM_004821	HAND	TGAGAGCAAGCGGAAAAGG	TCGGCTCACTGGTTTAACTC	mesoderm markers
NM_005992	BRACHYURY	CGTGCAGCTAGAGATGAAGG	CATATAGTCGGCCATGGGATC	
NM_001001890	RUNX1	CCAGGTTGCAAGATTTAATGACC	TTTTGATGGCTCTGTGGTAGG	hematopoietic differentiation transcription factors
NM_001145661	GATA2	TTCAATCACCTCGACTCGC	GCTGTGCAACAAGTGTGG	
NM_001136154	ERG	TGTGCAAGATGACCAAGGAC	ACCGTGGAGAGTTTTGTAAGG	
NM_152739	HOXA9	AATGCTGAGAATGAGAGCGG	GGGTCTGGTGTTTTGTATAGGG	
NM_001008540	CXCR4	CTTCATCTTTGCCAACGTCAG	GGACAGGATGACAATACCAGG	
NM_001130145	YAP	GGCTAGACCCAAGGCTTGAC	GGCTGTTTCACTGGAGCACT	naïve state-induced gene
NM_002167	ID3	CTACAGCGCGTCATCGACT A	TCGTTGGAGATGACAAGTTCC	naïve state-specific genes
NM_003412	ZIC1	GCGCTCCGAGAATTTAAAG A	GTCGCTGCTGTTAGCGAAG	
NM_006521	TFE3	TGCCTGTGTCAGGGAATCT G	CGACGCTCAATTAGGTTGTGAT	
NM_031944	MIXL1	AGCTGCTGGAGCTCGTCTT	CGCCTGTTCTGGAACCATAC	primed state-specific genes
NM_005442	EOMES	CGCCACCAAACTGAGATGA T	CACATTGTAGTGGGCAGTGG	

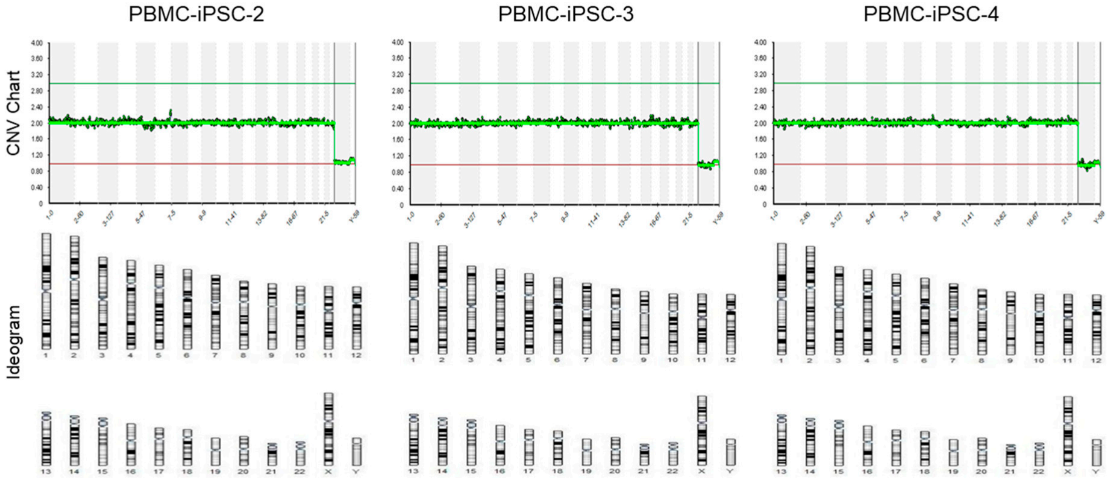

Supplementary Figure 1. Chromosome abnormalities of PBMC-hiPSCs.

Supplementary Figure 2. List of names, subclasses, and structures of tested flavonoids with their effect on stem cell proliferation.

Name	Subclass	Structure	Proliferation	Name	Subclass	Structure	Proliferation
Ipriflavone	Isoflavone	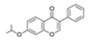	+++	Troxerutin	Flavone	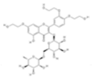	+++
Puerarin	Isoflavone	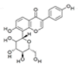	+++	3,2′-DHF	Flavone	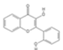	+++
Biochanin A	Isoflavone	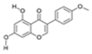	++	Neohesperidin	Flavanone	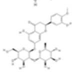	++
Icariin	Flavonol	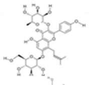	++	Naringenin	Flavanone	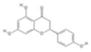	++
Rutoside	Flavonol	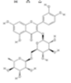	++	Hesperetin	Flavanone	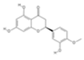	+++
Quercetin	Flavonol	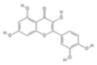	-	Hesperidin	Flavanone	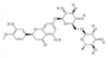	+++
Diosmetin	Flavone	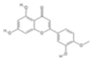	++	Methyl-Hesperidin	Flavanone	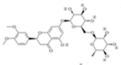	++
Apigenin	Flavone	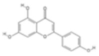	+	Naringin	Flavanone	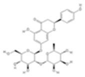	++
Baicalein	Flavone	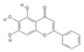	-	Taxifolin	Flavanone	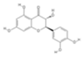	+++
Baicalin	Flavone	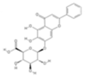	-	Naringin Dihydrochalcone	Flavanone	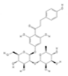	+++
Chrysin	Flavone	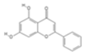	+++	Dihydromyricetin (Ampelopsin)	Flavanol	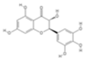	+++
Formononetin	Flavone	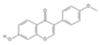	++	Bergenin	Flavanol	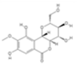	+
Kaempferol	Flavone	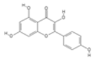	+++	Isoliquiritigenin	chalcone	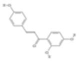	-
Luteolin	Flavone	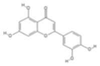	+	Butein	chalcone	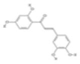	-
Myricetin	Flavone	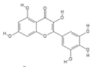	++	Phloretin	Anthocyan	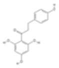	+++
Myricitrin	Flavone	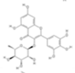	++	Silibinin	flavonolignan	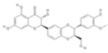	++
Nobiletin	Flavone	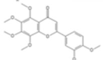	++	Silymarin	flavonolignan	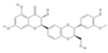	++
Phlorizin	Flavone	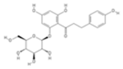	+++	Morin Hydrate	Flavone	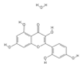	++
Quercetin Dihydrate	Flavone	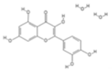	-	NHDC	Chalcone	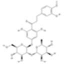	++
Tangeretin	Flavone	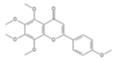	++				

Proliferation inhibition : -, proliferation no effect : +, proliferation low enhance : ++, proliferation high enhance : +++

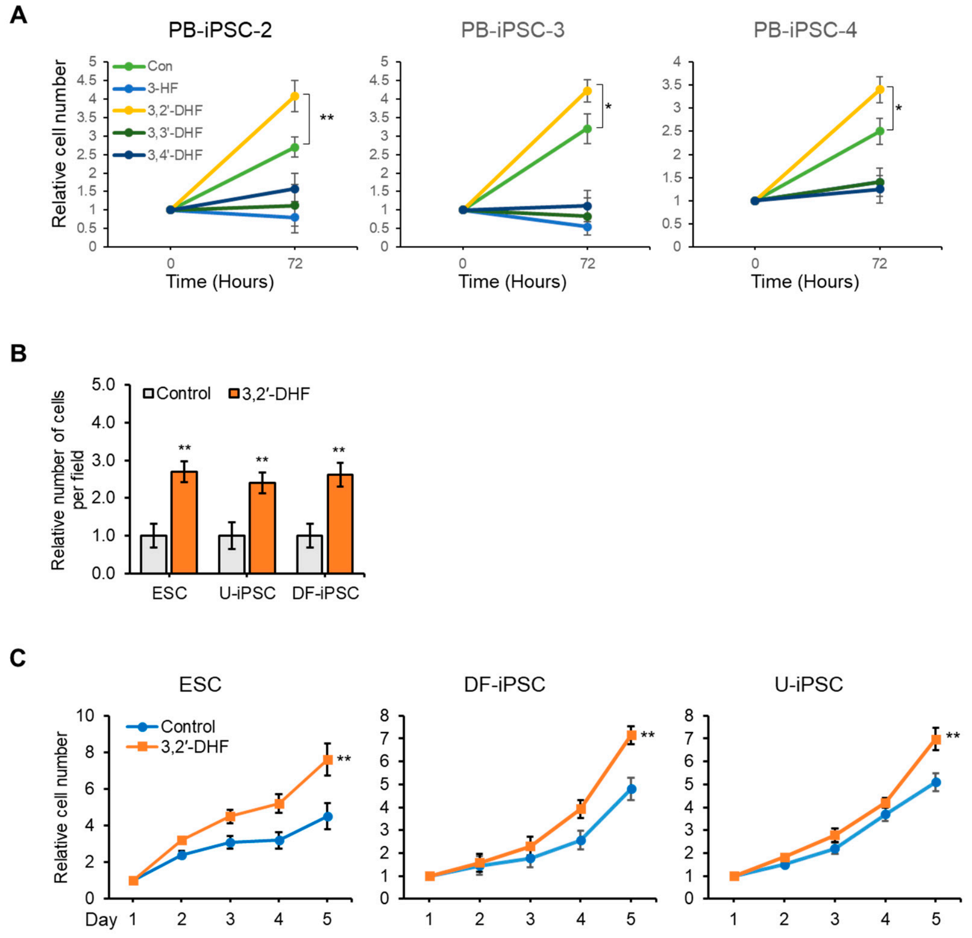

Supplementary Figure 3. (A) Proliferation effects of 10 μM 3,2′-DHF treatment on PB-iPSC lines. (B) Survival upon dissociation-induced apoptosis condition on several types of hPSCs. (C) Proliferation effect of 10 μM 3,2′-DHF treatment on several types of hPSCs. (* $p < 0.05$, ** $p < 0.01$). $n = 3$ biological samples.

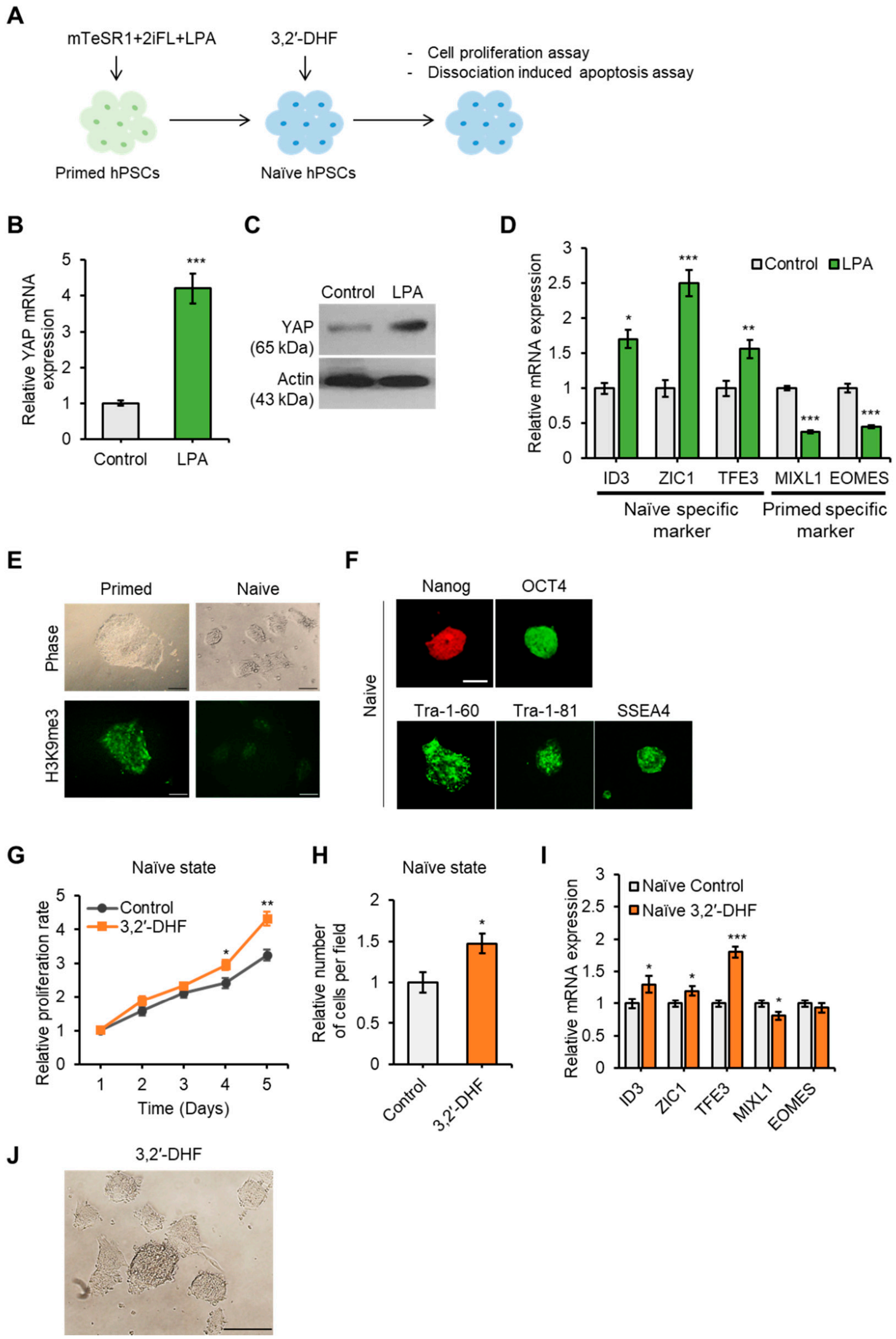

Supplementary Figure 4. Derivation of naïve state hiPSCs. (A) Scheme of conversion to naïve state hiPSCs. (B) mRNA expression of YAP. (C) Protein expression of YAP. (D) mRNA expression of naïve and primed state-specific markers. (E) Primed-specific flat and naïve state-specific dome-like colony morphology. H3K9me3 expression was more strongly reduced in naïve state hiPSCs than in primed state hiPSCs. Scale bar: 200 μm. (F) Cells with naïve state-specific dome-like colony morphology showed strong positive immunocytochemical staining for pluripotency markers Nanog, OCT4, Tra-1-60, Tra-1-81, and SSEA4. Scale bar: 100 μm. (G) Relative hiPSC proliferation rate with or without 3,2′-

DHF in the naïve state (H) Survival rate of naïve state hiPSCs upon dissociation-induced apoptosis with or without 3,2′-DHF. (I) Comparison of control and 3,2′-DHF mRNA expression of primed and naïve state-specific markers. (J) Naïve state-specific dome-like colony morphology of hiPSCs with 3,2′-DHF. Scale bar: 100 μm (* $p < 0.05$, ** $p < 0.01$, *** $p < 0.001$). $n = 3$ biological samples.

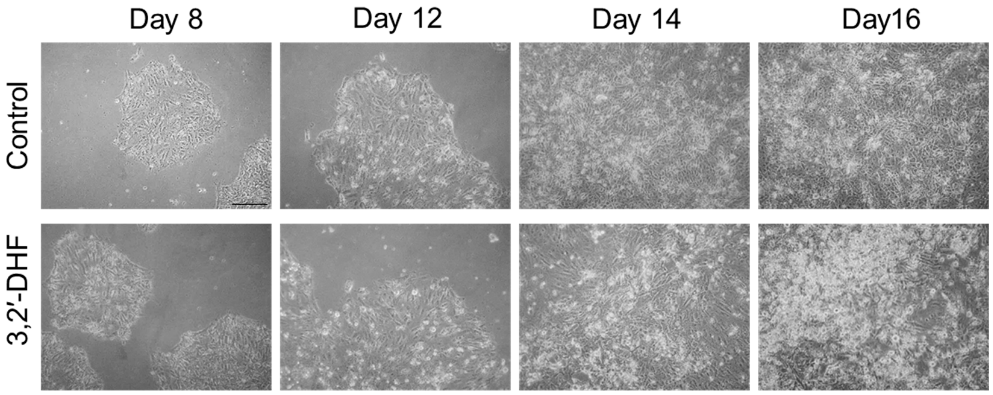

Supplementary Figure 5. hiPSC-derived HPC differentiation morphology at days 8, 12, 14 and 16 with or without 3,2′-DHF. Scale bar: 200 μm.
